# Validation of a Food Propensity Questionnaire for the Hellenic National Nutrition and Health Survey (HNNHS) and Results on This Population’s Adherence to Key Food-Group Nutritional Guidelines

**DOI:** 10.3390/nu12061808

**Published:** 2020-06-17

**Authors:** Theodoros Smiliotopoulos, Emmanuella Magriplis, Antonis Zampelas

**Affiliations:** Department of Food Science and Human Nutrition, Agricultural University of Athens, Iera Odos 75, 118 55 Athens, Greece; teosmiliotopoulos@gmail.com (T.S.); emagriplis@aua.gr (E.M.)

**Keywords:** dietary food records, self-reported food propensity questionnaire, validity

## Abstract

Background: Food propensity questionnaires (FPQs) are means of dietary assessment in nutritional epidemiology, which provide valuable information for long term intakes and food group consumption. These tools, however, may be subjected to misreporting and need to be validated against standard quantitative methods. Aim: The aim of this study was to examine the validity of the qualitative FPQ developed to assess the dietary habits of the general population in Greece during the Hellenic National Nutrition and Health Survey (HNNHS) and to assess the population’s intake of specific food groups in relation to guidelines. Methods: Validation analysis was based on 3796 [1543 men (42.82%) and 2253 women (57.18%)] participants of the HNNHS in relation to two interviewer-administered 24 h recalls (24 hR). Participants were asked to report the frequency of their dietary intake, using the FPQ provided. Correlations and significance between methods were assessed via Spearman correlation and a Two-sample Wilcoxon rank-sum (Mann-Whitney) test, respectively. Agreement between the FPQ and the 24 hR was performed using the Bland–Altman test and differences between the FPQ’s shown intakes and the recommended intakes by the Dietary Guidelines for Greek Adults were calculated. Results: Medium to weak correlations, but statistically significant (*p* < 0.05), were observed for most food groups between 24 hR and the FPQ; medium for fruits, dairy products, drinks, and spirits (ranging from ρ = 0.371 to ρ = 0.461; highest for drinks and spirits) and weak for vegetables, meat, fish, eggs, starch foods, sweets, nuts, fats and oils, and fast food (ρ = 0.111 to ρ = 0.290; lowest for starch foods). A non-significant correlation was found for legumes (ρ = 0.070). The mean intake agreement (Bland–Altman analysis) between the FPQ and the 24 hR was 96.08% and ranged from 94.43 to 99.34% for the 14 food groups under examination. When food group intakes were compared to guidelines, results showed that the population’s dietary intake was below the guidelines for fruits, vegetables, whole grains, fish, and legumes. On the other hand, it was above the guidelines for most of the “unhealthy” food groups, in particular, fast food, sweets, drinks and spirits, red meat, and sweets. Conclusions: The specific FPQ provides valid information on major food groups and can be used to examine long term dietary patterns in nutritional studies. Ιn addition, dietary intakes of Greek adults are problematic and initiatives at the public health level are necessary.

## 1. Introduction

Long term “unhealthy” nutritional habits have been associated with many adverse health outcomes [1] of public health concern, with countries worldwide being affected by malnutrition; an umbrella term that includes overnutrition, undernutrition, and “hidden hunger”, defined as micronutrient related malnutrition [1]. Validated tools for measuring dietary intake in populations is therefore warranted and should be population specific [2]. The most common dietary assessment methods used in dietary surveys are the Food Frequency Questionnaires (FFQs) and the 24 h recalls (24 hR) [2,3,4].

Both methods have advantages and disadvantages and each provides valuable information when they are in agreement. The 24 hR is the gold standard for estimating energy, macro- and micro-nutrient intake, however it cannot be used to evaluate frequency of intake, hence this can lead to over-or under-estimation over a longer period of time. It also lacks the ability to assess episodically or periodically consumed foods [5]. Food frequency and sporadic food consumption can be evaluated using FFQs and are able to provide important information on food intake frequencies over longer periods of time. These lack, however, precision in portion size estimation compared to 24 hR, but can be used as an important covariate in supplementing multiple recalls for estimating usual intake of food groups [3,6]. Considering these facts as well as the potential of under-reporting of food intake, careful evaluation of tools used in population dietary assessment is needed. The level of simplicity of a FFQ is of great importance as there should be no loss of information [7,8] in order to unveil the actual relation between food intakes and dietary patterns with health outcomes [9]. This has led many researchers to combine food frequency questionnaires (FFQs) with 24 hR (usually two) to assess dietary habits and patterns, following tool validation.

The Food Propensity Questionnaire (FPQ) was introduced in 2006, which is considered quite recent in science [6], as a method to estimate usual long-term food intake. It is a qualitative FFQ that can be used along with the 24 hR in order to estimate long term food intake, addressing variation and frequency. This questionnaire, however, needs to be modified in order to account for local foods, food culture, and behaviour based on literature of the studied population [2]. The modifications need to be correctly validated against usual dietary intakes (24 hR), accounting not only for correlation between foods, but agreement as well [8,10] in order to avoid false positive results [10]. Results from these two dietary methods can be used to assess a population’s adherence to dietary recommendations.

Therefore, the aim of this study was to examine the validity of the qualitative FPQ developed to assess dietary habits of the general population in Greece during the Hellenic National Nutrition and Health Survey (HNNHS) and to assess the population’s intake of specific food groups in relation to guidelines using the FPQ and 24 hR.

## 2. Methods

### 2.1. Study Population

The Hellenic National Nutrition and Health Survey (HNNHS) includes a nationally representative sample. Details of the study design, participant selection, and questionnaires administration have been described elsewhere [11,12]. Briefly, the total sample of HNNHS consisted of 4574 participants aged ≥6 months that resided in Greece. Pregnant and/or breastfeeding women and people who lived outside a private household (e.g., military service, hospitals, institutions) were excluded. A multi-stage stratified design was used, based on the 2011 census data. Sample selection was performed upon assessing geographical density in 54 prefectures, as well as age group and sex distribution in each area. Individual selection was performed at the household level, with only one individual per age group being eligible and no more than two per household. Data collection was conducted between September 2013 and May 2015. All participants (or legal guardians) signed a consensus document before entering the survey. All work was carried out upon obtaining individual consent and was approval by the Ethics Committee of the Department of Food Science and Human Nutrition of the Agricultural University of Athens and by the Hellenic Data Protection Authority (HDPA).

The final sample consisted of 3796 adult participants (42.82% men). Mis-reporters, including under- and over-reporters, were defined as those reporting energy intake <500 and >6000 kcal/day (4% of the sample) and were excluded from the final analysis. Mis-reporters were also identified by the modified Goldberg equation [13,14] (35.6% of the sample), but were included in the main analysis, in accordance with the recommendations of the European Food Safety Authority (EFSA) [15], to avoid systematic error by excluding individuals that may be on a restricted diet or consume extremely large quantities for any reason; psychosomatic or other.

### 2.2. Dietary Assessment

Methods of dietary assessment were also chosen as per European Food Safety Authority (EFSA), recommendations for the harmonization of data across countries’ member states of the European Union [16]. More specifically, FOODEX2 was used to code and categorize all foods.

The Computer Assisted Personal Interview (CAPI) method was used for data collection by trained personnel during which the first 24 hR was performed. This was conducted at the participant’s residence using the latest version of the USDA Automated Multiple-Pass Method (AMPM) [17], a method that increases reporting accuracy using a five-step process [17]; the first is unstructured and uninterrupted, during which the participants list all foods and beverages consumed the previous day, in any order that they remember them. The next three steps use a structured approach, including use of memory cues to help the participants remember consumption details. The last step is called the Final Probe Step, where the interviewer uses extra cues to assure that the participants have listed all foods and beverages consumed. Finally, in order to achieve accurate portion sizes, specific grids and household volume measures (glasses, cups, plates, various spoon sizes, etc.), as well as age-specific specific food atlases were used. Copies of these food atlases were given at the end of the interview to the participants for them to use in the second 24 hR, which was conducted via phone. The phone interview was performed on a different and non-consecutive day, 8 to 20 days following the first. The completed FPQs were then collected at the mobile centers where participants came for measurements or the interviewers revisited the participants’ residence to obtain them. These were checked for completion and the participant was asked for clarifications if needed.

In order to assess frequency of consumption and capture the whole diet over a longer time interval (one year on average), a food propensity questionnaire (FPQ) was also developed and used in HNNHS. This FPQ was designed following International and European recommendations. More specifically, it was based on the Diet History Questionnaire (DHQ) [18], used by the National Health and Nutrition Examination Survey (NHANES), developed by the National Institute of Health (NIH), and the FPQ used by the pilot study of a Pan-European dietary survey (PILOT-PANEU) [19,20,21]. A total of 86 foods were included, based on the food list recommended by PILOT-PANEU and with foods consumed by the Greek population as per data derived from the Data Food Networking [22] and from Food Balance Sheets provided by the Food & Agricultural Association [23]. Foods were grouped and listed in 10 categories, as recommended by Willet et al. [5] and were organized according to two large population studies; the Nurses’ Health study and the Health Professionals’ Follow up Study. More specifically, and in order to be listed, the groups were the following: dairy products, bread-cereal & other carbohydrates, fruit, vegetables, eggs, meat (including processed meat products, game, snails, and poultry), fish & seafood, water & beverages, olives, fats & oils, sweets, and baked goods & nuts. These food groups are shown in greater detail in Table 1. For the bread-cereal group & other carbohydrate group, other than potatoes, whole wheat products were distinguished from their processed, white counter product. Fried potatoes were also distinguished. The FPQ can be viewed in detail in the Supplementary File. Portion size was not included in the FPQ since data from this questionnaire were not intended to be used in energy and macronutrient estimation, but to examine frequency of food group intake. In addition, a total of 11 supplementary food questions were included in order to get more detailed information on foods; for example, type of oil or fat usually used during cooking and how often fish consumed is fried. All additional questions can also be seen in the Supplementary File. Printed FPQs were given to all participants at the end of the interview process and they were asked to complete them in their own time. They were provided analytical instructions with examples on completing the questionnaire and were asked to report their frequency of using foods in reference to the preceding year in order to account for seasonal variation. Ten potential response options were given to accurately capture frequency of intake: never, <1 time per month, 1–3 times per month, 1 time per week, 2–4 times per week, 5–6 times per week, 1 time per day, 2–3 times per day, 4–5 times per day, and ≥6 times per day.

In order to evaluate dietary intake compared to recommendations, the grains & cereals and meat food groups were desegregated to be compared with nutritional recommendations. Specifically, meat was separated into (i) red meat, game & processed meat products, and (ii) white meat including poultry and snails. Grains were separated into whole grain cereals and refined.

The food classification and description system developed by the European Food Safety Authority (EFSA) [23] was used for the standardized characterization of the reported food items. The Nutrition Data System for Research (NDSR; Nutrition Coordinating Center, University of Minnesota, USA) was used to assess the nutrient content of the reported food items [24], with the Greek food composition tables for traditional recipes adding to the result [11,12]. Food items were divided into 30 food groups based on nutrient composition and culinary use. Mixed dishes (e.g., sandwich, salad) and recipes (e.g., lasagna, moussakas) were disaggregated into their ingredients, which were then proportionally assigned to the appropriate food groups. At this point, it is important to mention that no disaggregation was performed for pizza, hot dogs, burgers, and souvlaki, which were categorized directly as fast-food, due to their high fat and sodium content and their low nutrient quality. Consequently, the assessed meat and cereal intakes calculated do not include red meat from souvlaki and hamburgers, which are key ingredients, and dairy does not include cheese found on pizza. Food items, however, had to be categorized into one food group and these items were included in the non-recommended “fast-food” category. The format g/day was used to represent intake for all foods and beverages. Mean intakes were calculated for total energy and macronutrients, using both recalls. Only one recall was used for 606 individuals (15.5% of the sample), since they had refused to provide a second during data selection. These individuals were included in the final analysis upon examining potential reporting bias, meaning potential significant variation in total energy and macronutrient intakes between individuals who provided one recall and those who provided two. No significant differences were found.

Frequency of consumption was translated into servings per day by dividing mean reported frequency by the total days (for example, 1 to 3 times per month: mean 2 times per month divided by 30 = 0.067 servings per day, and once a week = 1/7 = 0.143 servings per day). This was then multiplied by the grams that were quantified from the two 24 hR to obtain a relative intake over time and to decrease variability. Mean intake from FPQ was calculated by multiplying the FPQ’s reported frequency with the average portion sizes that are given in the Dietary Guidelines for Greek Adults [25]. For example, if bread was consumed 5–6 days per week (and portion size = 68.75 g), that was converted to 5.5 times per week, which means that it was consumed 0.79 times per day, which is approximately 54 g per day. This was done in order to assess the degree of agreement between the two dietary assessments and not only the correlation. The final analysis included the quantification of the usual intake from the FPQ, with the amounts reported in the 24 hR as recommended by EFSA [26].

### 2.3. Anthropometry

Body Mass Index (BMI) was calculated in kg/m^2^ from reported body weight in kg and height in meters.

### 2.4. Statistical Analysis

Variables were checked for normality by the use of histograms, P-P plots, and k-density. Descriptive statistics used included percentages for categorical, means and standard deviations (SD) (for normally distributed), and median and quartiles (25–75%) for quantitative skewed variables. Comparisons of participant’s mean food intake between FPQ and 24 hR was performed using the two-sample Wilcoxon rank-sum (Mann-Whitney) test and the Spearman’s correlation coefficient (ρ or r_o_), to account for skewness. Spearman’s correlation coefficient was used to evaluate the level of agreement between the FPQ and the gold standard of the 24 hR, stratifying by sex or weight status. The strength can be assessed by these general guidelines, in absolute numbers: a. 0.1 < |ρ| < 0.3: small/weak correlation; b. 0.3 < |ρ| < 0.5: medium/moderate correlation; c. 0.5 < |ρ|: large/strong correlation.

The Bland–Altman (B&A) method [27] was used to determine the level of agreement between the mean FPQ and the 24 hR, as was recommended by these, two world known statisticians, for adequate assessment between two quantitative measurements. This method constructs limits of agreement by using the mean and the standard deviation (sd) of the differences between two measurements. The graph that results from this approach is a scatter plot XY, in which the Y axis shows the difference between the two paired measurements (A − B) and the X axis represents the average of these measures ((A + B)/2) [27,28]. It is recommended that 95% of the data points should lie within ± 2 sd of the mean difference, but generally, if more than 90% of the data points lie within ±2 sd, then the results seem to be in agreement [16]. Therefore, levels of agreement between the difference of the 24 hR record and the FPQ (in grams/day) were considered the interval [average difference in dietary intake ± 1.96 standard deviation of the difference] and as suggested by the authors [28]. Statistical significance was considered when *p*-values < 0.05. The software that was used for data entry and statistical tests of hypotheses was STATA version 14.0 (STATA/MP 14.0 for Windows, STATA Corp., USA).

## 3. Results

The distribution of the main characteristics of the study’s sample is presented in Table 2. The analysis sample consisted of 3796 participants (males *n* = 1543, 42.82% and females *n* = 2253), with a median age of 39 and upper and lower quartiles of 57 and 27 years, respectively. Statistically significant differences were observed by sex between weight (*p* < 0.001) and height (*p* < 0.001) of the two subgroups of the study’s population, but not in relation with age (*p* = 0.289) and BMI (*p* = 0.819).

Table 3 gives the distribution for the consumption of 14 food groups assessed through the FPQ and the 24 hR food records and the results from the Two-sample Wilcoxon rank-sum (Mann-Whitney) test and Spearman’s correlation coefficients. In the Two-sample Wilcoxon rank-sum (Mann-Whitney) test, *p*-values refer to the examination of whether there are statistically significant differences between variables that are present in the table. Statistically significant differences were observed between the results of the FPQ and the 24 hR for all the food groups (*p* < 0.001), except for the food group of starch foods (*p* = 0.974). Moreover, Table 3 displays the results of the additional validation analysis of the FPQ through the calculation of the Spearman’s correlation coefficient. Medium to weak correlations, but statistically significant (*p* < 0.05), were observed for most food groups between 24 hR and the FPQ; medium for fruits (ρ = 0.373), dairy products (ρ = 0.371), drinks & spirits (ρ = 0.461) and weak for vegetables (ρ = 0.136), meat (ρ = 0.268), fish (ρ = 0.166), eggs (ρ = 0.142), starch foods (ρ = 0.111), sweets (ρ = 0.261), nuts (ρ = 0.290), fats and oils (ρ = 0.144), and fast food (ρ = 0.259). A non-significant correlation was found for legumes (ρ = 0.070).

Table 4 shows Spearman’s correlation coefficients for Validity of FPQ versus 24 hR Dietary Records, by Subgroups of Participants (men, women, normal weight people, overweight people, and obese people) and it can be seen by the results that the power of the correlations is nearly the same as in Table 3. The main differences are that there is a non-significant correlation in the consumption of vegetables of normal weight people (ρ = 0.090) and of starch foods for men (ρ = 0.097), a weak correlation in the consumption of legumes for men (ρ = 0.121), a medium correlation in the consumption of nuts for women (ρ = 0.307) and normal weight people (ρ = 0.311), and there is a strong correlation in the consumption of drinks and spirits for overweight people (ρ = 0.500).

According to the Bland–Altman analysis, the mean intake agreement between the nutrition assessment method of the FPQ and the 24 hR was 96.08% and ranged from 94.43% to 99.34% for the 14 food groups under examination (Table 5). In particular, Table 5 shows that on the basis of the Bland–Altman analysis, the agreements between the two nutrition assessment methods are the following: fruits (95.60%), vegetables (96.40%), dairy (95.79%), meat (96.60%), fish (96.65%), eggs (96.12%), starch foods (95.69%), legumes (96.86%), sweets (94.78%), beverages (94.43%), nuts (99.34%), fats and oils (96.61%), drinks and spirits (95.15%), and fast food (95.47%).

**Figure 1:**
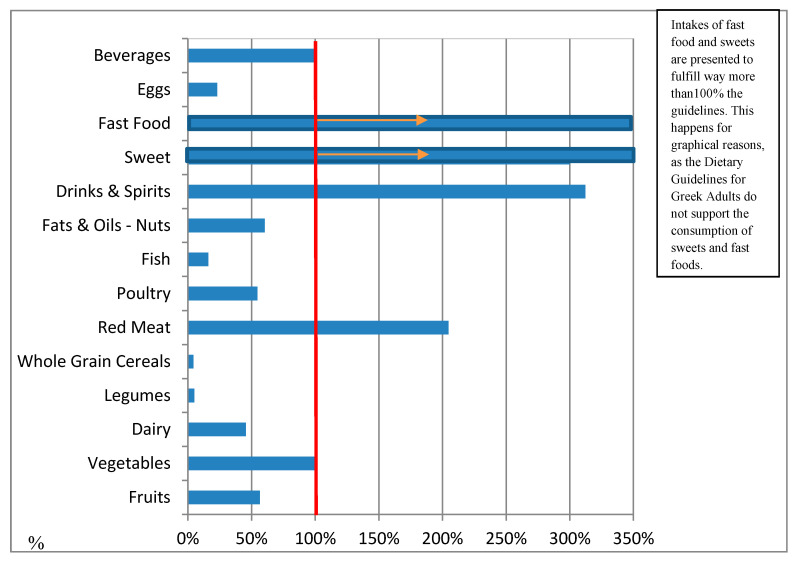
Combined 24 hR with FPQ’s intakes of Greek males and agreement with the Dietary Guidelines for Greek Adults. The percentages of agreement were calculated from the combined intakes (grams/day) of food groups in relation to reported frequency of intake (FPQ) and reported amount of intake (24 hR) of Greek males and the Dietary Guidelines for Greek Adults. The red line represents the point at which guidelines are fulfilled.

**Figure 2:**
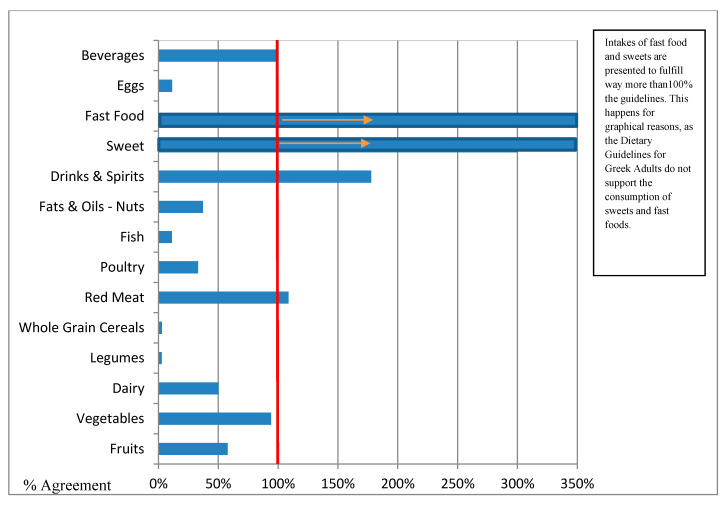
Combined 24 hR with FPQ’s intakes of Greek females and agreement with the Dietary Guidelines for Greek Adults. The percentages of agreement were calculated from the combined intakes (grams/day) of food groups in relation to reported frequency of intake (FPQ) and reported amount of intake (24 hR) of Greek females and the Dietary Guidelines for Greek Adults. The red line represents the point at which guidelines are fulfilled.

Last, but not least, Table 6 presents the mean consumption for each of the food groups according to the intakes shown by the 24 hR and the combined intakes that result when 24 hR is used in combination with the FPQ. This table also presents the % agreement between the aforementioned intakes and the recommended ones by the Dietary Guidelines for Greek Adults [25]. In particular, according to the Dietary Guidelines for Greek Adults, the suggested daily consumption for every food group is: 480 g for a variety of fruits, 700 g for a variety of vegetables, 334 g for dairy products, 75 g for legumes, 220 g of refined cereals and 227 g of whole grain cereals, 19.29 g of red meat, 28.93 g of poultry, 54 g of fish, 112.5 g of fats, oils, and nuts, 30 g to 60 g of drinks (for males and females, respectively) and spirits, 29 g of eggs, and 2250 g of water and beverages. Additionally, the consumption of sweets and fast food is not recommended and it is suggested that these food groups should be avoided. On this basis, it can be seen that according to the 24 hR, the guidelines were % fulfilled as follows: 30.98% for fruits, 27.69% for vegetables, 42.18% for dairy products, 19.63% for legumes, 29% for refined cereals and 39% for whole grain cereals, 266% for red meat, 107% for poultry, 43.30% for fish, 24.54% for fats, oils, and nuts, 130.79% for drinks and spirits, 36.28% for eggs, and 48.74% for beverages. The consumption of fast food and sweets exceeds the guidelines. Moreover, according to the combined results (FPQ’s frequency × 24 hR amount of consumption), the consumption for each food group is as follows: 270.7 g of fruits (56% of the suggested portion), 632.6 g of vegetables (90% of the recommended intake), 2312 g of dairy products (69% of the recommended intake), 2.57 g of legumes (3% of the recommended intake), 132 g of refined cereals (60% of the recommended intake) and 7.17 g of whole grain cereals (3% of the recommended intake), 27.7 g of red meat (144% of the recommended intake), 11.30 g of poultry (39% of the recommended intake), 6.75 g of fish (13% of the recommended intake), 48.9 g of fats, oils, and nuts (44% of the recommended intake), 92.2 g of drinks and spirits (13% of the recommended intake), 4.76 g of eggs (16% of the recommended intake), 430.6 g of beverages (86% of the recommended intake), and the consumption of fast foods and sweets exceeds the guidelines (59.04 g of sweets and 33.5 g of fast food). Finally, in Figure 1 and Figure 2, the bar chart illustrates the percentage of males and females that meet or exceed the aforementioned guidelines, respectively. The red line signifies the limit of meeting the Dietary Guidelines for Greek Adults. In these figures, it can be seen that both men and women surpass recommendations for red meat (39.49 g per day for men versus 20.98 g per day for women), fast food, sweets, and drinks & spirits, whereas whole grain cereals and legumes are minimally consumed (<10% of recommended intake). Men overall consumed more red meat, poultry, fats, oils and nuts, drinks and spirits, fast food, and eggs than women, while for the rest of the examined food groups, consumptions seem to be more or less the same.

## 4. Discussion

In the present study, the validity of the qualitative FPQ used in the HNNHS for the general population was tested against a two non-consecutive day 24 hR. The analyses showed over 90% agreement between the two methods in all the primary food groups and beverages consumed by participants and specifically, the level of agreement (using the Bland–Altman method) ranged between 94.14% to 99.37% for the consumption of fruit, vegetables, dairy, meat, fish, eggs, starch foods, legumes, sweet, beverages, nuts, fats-oils, drinks-spirits, and fast food. Validation was confirmed in all groups, defined by sex and by weight status (normal weight, overweight, and obese), supporting the premise that the FPQ can be useful as a predictor of the probability of consumption for the general population of the Greek adults and supplement recall data collected in national surveillance. The study also revealed an “unhealthy” dietary pattern followed by Greek adults. More specifically, our results showed that the consumption of foods that are categorized as “healthy” is low (fruits, vegetables, legumes, whole grain cereals, poultry, fish, and eggs) and the consumption of “unhealthy” foods is high (red meat, drinks and spirits, sweets, and fast food).

Dietary assessment is very important for nutritional epidemiology in order to retrieve associations between diet and health outcomes. Τhe most established dietary assessment methods to accomplish this task are the FFQs (in studies assessing diet and disease associations) and the 24 hR (mostly in nutrition surveillance studies) [3,4]. For both of these methods, sex can be a confounding factor, referring to the accuracy of the results [29]. What is more, the combination of these two methods has become increasingly popular during the last few years. The use of multiple 24 hR (usually two per participant) in conjunction with a non-quantitative FFQ (stated mostly as food propensity questionnaire—FPQ) can estimate and withdraw the effects of within-person variation in dietary intake. In such studies, a statistical model is usually applied to assure that the correlation between the probability of consuming a food (as reported in the FPQ) and the amount consumed on a particular day (as reported in the two 24 hR) can be observed and further incorporates co-variate information relating to 24 hR [4,10]. The aforementioned facts increase the importance of the present study as it opens up the way of using such a methodology in epidemiological studies referring to the Greek population. To date, many FFQs have been developed and validated for ethnic groups [30,31,32], with their main purpose as the assessment of the association between dietary factors and various health conditions or general public health, but in reference to the Greek general population, none of them has been tested or validated. Validation of such an FFQ is of great importance as an accurate assessment of the dietary intake and habits of Greece’s population can result in great decisions concerning public health and wellbeing. A combination of at most two 24 hR and an FFQ has been used in many validation studies reporting a strong correlation between the assessment tools [29,32,33,34].

The present study evaluated the FPQ which would be used in the HNNHS in order to assess dietary habits of Greece’s general population. In general, moderate to weak correlations were observed and, in a few cases, the correlation was strong in reference to the examined food groups, between the FPQ and the 24 hR. Moreover, validity was tested not only by the use of Spearman’s correlation coefficients, but also by measuring the agreement between the FPQ and the 24 hR. This strengthens the validity of the results as correlation coefficients can mislead the results into false assumptions [10]. The use of the Bland–Altman method assesses the agreement between the methods and is more suitable to the use of correlation coefficients and adds considerably to the results [10]. Additionally, another aspect that adds to the importance of this study is the fact that agreement between dietary habits of a Greek population’s representative sample and Dietary Guidelines for Adults [25] was examined. Results were also in agreement for both sexes, although men were found to have slightly stronger agreement in all food groups compared to women. Moreover, validity remained high (higher than 94%) for all the food groups and beverages. These results are in agreement with those reported by previous studies [16,32,35,36,37,38]. On the other hand, a limitation of this study is that this FPQ is a qualitative means to assess dietary intake and cannot be used to assess energy and macronutrients intakes quantitatively if used alone. It is, however, a valuable tool for all adults, of both sexes and irrespective of weight status, as shown in this paper. Specific age groups, however, were not examined, since this was not the aim of this validation study. It should also be mentioned that this FPQ was designed to address the major food group consumption of the Hellenic population. Modifications and consequently additional validation may be necessary if it is to be used for other populations.

The results of the present study also show that the dietary intake of Greek people does not adhere to nutritional recommendations. They tend to overconsume most of the non-recommended food groups and fall short when it comes to the recommended ones. It must be underlined that red meat intake was above recommendations, although calculated intake did not include red meat found in souvlaki and in burgers (since those were evaluated as fast food). Refined grains were also high, surpassing the mid recommended levels (60%), although pita bread, pizza dough, and hamburger buns were not included in the analysis. The results agree with those reported in other epidemiological studies referring to the Greek population. The European Prospective Investigation into Cancer and Nutrition (the EPIC project), which was conducted for a sample of 20,882 Greek people aged 25 to 86 years old, showed that the consumption of fruits, vegetables, cereals, dairy products, legumes, fish, eggs, oils, and nuts was below the recommendations and above for red meat and drinks and spirits [39]. Another study, the ATTICA study, which was conducted with a sample of 3042 adults, showed that the consumption of fruits, red meat, oils, nuts, drinks, and spirits was above the recommendations and the consumption of vegetables, cereals, dairy products, legumes, poultry, fish, and eggs was below [40]. The EPIC Study showed that among the other nine countries which participated in the study, Greece had one of the highest consumption in vegetables and legumes, but the consumption of red meat, poultry, fish, eggs, and drinks and spirits was above the European average. In addition, the consumption of fruits, cereals, and dairy products was below the European average [41]. Finally, after comparing food consumption patterns in the 1960s from the Seven Countries Study, it was reported that modern Greek people have reduced the consumption of legumes and olive oil and at the same time, they have increased the consumption of meat and cheese [42].

## 5. Conclusions

The information gathered from FPQ used to assess the nutritional intake of a representative sample of the Greek population in the HNNHS was found to be an efficient tool, when compared to reported 24 hR, and can be used in the future to examine long term dietary patterns. Also, the use of the FPQ in combination with two 24 hR revealed an unhealthy dietary pattern followed by Greek adults, charactarized by low consumption of fruits, vegetables, whole grains, legumes, poultry, fish, and eggs and high consumption of refined cereals, red meat, alcohol, sweets, and fast food, a pattern that substantially differs from the Mediterranean diet.

## Figures and Tables

**Table 1:** Basic Foods and beverages included in the food propensity questionnaire (FPQ) used.

Food or Beverages	Frequency of Consumption Measured
Fruits (orange, apple, pear, etc., juices)	Never, <1 per month, 1–3 per month, 1 per week, 2–4 per week, 5–6 per week, 1 per day, 2–3 per day, 4–5 per day, 6 + per day
Vegetables (tomatoes, cucumber, lettuce, cabbage, etc.)	
Dairy (milk, yoghurt, cheese, milk substitutes, cream)	
Meat (red meat, including poultry, game, snails, and processed meat products)	
Fish (tuna, sardine, octopus, calamaries etc.)	
Starch foods (cereals, bread, pasta, rice)	
Legumes (beans, fava, lentils, etc.)	
Fats and Oils (butter, margarine, olive oil, canola oil, etc.)	
Nuts (walnuts, almonds, peanuts, etc.)	
Alcoholic beverages, wines and spirits (beers, red or white wines, whiskies, vodkas, etc.)	
Sweet (sweet beverages, sweet desserts, sweeteners, sugars, baked sweet products, sweet puff pastry, compote)	
Eggs	
Fast food (salty snacks, dressings, sauces, fast food, salty or sweet pies, salty puff pastry)	
Beverages (water, regular, sugar free, carbonated or non-carbonated)	

**Table 2:** Anthropometric variables of the participants.

Variable	Sample (*n* = 3796) ^1^	Men (*n* = 1543, 42.82%)	Women (*n* = 2253, 57.18%)	*p*-Value ^3^
Age ^2^ (years)	39 (27, 57)	37 (27, 56)	40 (26, 58)	0.289
Weight (kg)	72 (61, 82.5)	82.48 ± 13.76	64 (57, 73)	<0.001
Height (m)	1.69 ± 0.11	1.77 (1.72, 1.82)	1.64 (1.60, 1.68)	<0.001
BMI ^4^	25.41 ± 4.78	26.29 ± 4.04	24.80 ± 5.16	0.819

**^1^** The letter n represents the population of the study’s sample. **^2^** Variables are presented by the quartiles format 50 (25, 75) for variables that follow a non-normal distribution and by the mean ± sd format for the ones that follow a normal distribution. **^3^** *p*-values refer to the examination whether there are statistically important differences between variables that are present in the table, by sex. BMI was examined using the *t*-test method for two samples and for the rest of the variables, the Two-sample Wilcoxon rank-sum (Mann-Whitney) test. When *p* < 0.05, there is a statistically significant difference. **^4^** BMI (Body Mass Index) was calculated using the following: weight (kg)/height^2^ (m^2^).

**Table 3:** Distribution of the Consumption of the Foods and Beverages in the Validation Study and Results from the Wilcoxon non-parametric pairwise comparison test and Spearman correlation coefficients Analysis of Validity.

	24 hR	FPQ ^3^	Spearman Correlation Coefficients	Wilcoxon Non-Parametric Pairwise Comparison Test
Fruit	105 (0, 216.67) ^1,2^	205.12 (125.76, 413.44) ^1^	0.373 **	<0.001 ^4^
Vegetables	151.58 (71.90, 258.90)	580.48 (379.58, 861.18)	0.136 **	<0.001
Dairy	100.64 (32.28, 213.24)	254.09 (125.81, 386.57)	0.371 **	<0.001
Meat	53.82 (10.50, 111.83)	137.43 (80.60, 207.77)	0.268 **	<0.001
Fish	0 (0, 0)	37.80 (22.65, 61.80)	0.166 **	<0.001
Eggs	0 (0, 9.84)	7.15 (3.35, 21.45)	0.142 **	<0.001
Starch foods	121.89 (67.92, 205.62)	129.78 (94.54, 175.44)	0.111 **	0.974
Legumes	0 (0, 0)	64.75 (39.73, 100.10)	0.070 **	<0.001
Sweet	42 (7.50, 132.50)	75.14 (39.84, 128.88)	0.261 **	<0.001
Beverages	1444.74 (987.30, 1928.75)	1825.75 (1463.50, 2249.00)	0.251 **	<0.001
Nuts	0 (0, 0)	3.58 (0.43, 10.73)	0.290 **	<0.001
Fats & Oils	16.59 (6.82, 30.61)	37.80 (19.72, 52.38)	0.144 **	<0.001
Drinks & Spirits	0 (0, 95.19)	42.83 (11.31, 125.00)	0.461 **	<0.001
Fast Food	14.69 (0, 127.68)	63.32 (34.91, 117.55)	0.259 **	<0.001

** Correlation is significant at the 0.01 level (2-tailed). ^1^ Intakes are presented as g/day. ^2^ Variables are presented by the quartiles format 50 (25, 75) for variables that follow a non-normal distribution and by the mean ± sd format for the ones that follow a normal distribution. ^3^ FPQ, food propensity questionnaire. ^4^ *p*-values refer to the examination whether there are statistically significant differences between variables that are present in the table. Variables were tested using the Two-sample Wilcoxon rank-sum (Mann-Whitney) test. When *p* < 0.05, there is a statistically significant difference.

**Table 4:** Spearman’s correlation coefficients for Validity of FPQ ^2^ versus 24 hR, by subgroups of participants.

	Total Sample (*n* = 3796) ^1^	Men (*n* = 1543)	Women (*n* = 2253)	Normal Weight (*n* = 1653)	Overweight (*n* = 1329)	Obese (*n* = 624)
Fruit	0.373 ** ^3^	0.395 **	0.357 **	0.389 **	0.384 **	0.367 **
Vegetables	0.136 **	0.145 **	0.149 **	0.090 **	0.161 **	0.193 **
Dairy	0.371 **	0.356 **	0.379 **	0.368 **	0.378 **	0.363 **
Meat	0.268 **	0.290 **	0.231 **	0.279 **	0.261 **	0.247 **
Fish	0.166 **	0.194 **	0.142 **	0.206 **	0.171 **	-
Eggs	0.142 **	0.143 **	0.141 **	0.138 **	0.125 **	0.158 **
Starch foods	0.111 **	0.097 **	0.114 **	0.162 **	0.100 **	-
Legumes	0.070 **	0.121 **	0.045 **	0.090 **	0.088 **	-
Sweet	0.261 **	0.234 **	0.279 **	0.237 **	0.233 **	0.299 **
Beverages	0.251 **	0.184 **	0.286 **	0.294 **	0.167 **	0.268 **
Nuts	0.290 **	0.267 **	0.307 **	0.311 **	0.290 **	0.239 **
Fats & Oils	0.144 **	0.141 **	0.161 **	0.131 **	0.181 **	-
Drinks & Spirits	0.461 **	0.454 **	0.413 **	0.424 **	0.500 **	0.473 **
Fast Food	0.259 **	0.275 **	0.220 **	0.259 **	0.259 **	0.285 **

** Correlation is significant at the 0.01 level (2-tailed). ^1^ The letter n represents the population of the study’s sample. ^2^ FPQ, food propensity questionnaire. ^3^ Spearman’s correlation coefficient was used to determine the level of correlation (0.1–0.3: weak correlation, 0.3–0.5: medium correlation, and 0.5–1.0 strong correlation).

**Table 5:** Distribution of Consumption of the Foods and Beverages in the Validation Study and Results from the Bland–Atman Analysis of Validity *.

	FPQ ^1^	24 hR	% Within the Agreement Interval ^2^
Fruit	205.12 (125.76, 413.44)	105 (0, 216.67)	95.60
Vegetables	580.48 (379.58, 861.18)	151.58 (71.90, 258.90)	96.40
Dairy	254.09 (125.81, 386.57)	100.64 (32.28, 213.24)	95.79
Meat	137.43 (80.60, 207.77)	53.82 (10.50, 111.83)	96.60
Fish	37.80 (22.65, 61.80)	0 (0, 0)	96.65
Eggs	7.15 (3.35, 21.45)	0 (0, 9.84)	96.12
Starch foods	129.78 (94.54, 175.44)	121.89 (67.92, 205.62)	95.69
Legumes	64.75 (39.73, 100.10)	0 (0, 0)	96.86
Sweet	75.14 (39.84, 128.88)	42 (7.50, 132.50)	94.78
Beverages	1825.75 (1463.50, 2249.00)	1444.74 (987.30, 1928.75)	94.43
Nuts	3.58 (0.43, 10.73)	0 (0, 0)	99.34
Fats & Oils	37.80 (19.72, 52.38)	16.59 (6.82, 30.61)	96.61
Drinks & Spirits	42.83 (11.31, 125.00)	0 (0, 95.19)	95.15
Fast Food	63.32 (34.91, 117.55)	14.69 (0, 127.68)	95.47

* Results are presented as median and quartiles (1st, 3rd). ^1^ FPQ, food propensity questionnaire. ^2^ % within the agreement interval between the results of the FPQ and the 24 hR per food group. Bland and Altman analysis was used to determine the percentage of agreement between the two methods.

**Table 6:** Level of agreement between the Dietary Guidelines for Greek Adults and Greece’s population’s consumption per food group.

A/A	Food Group	DGG ^1^ (g)	24 hR ^2^ (g)	24 hR ^3^ %	Combined 24 hR—FPQ ^4^ Intake (g)	Combined 24 hR—FPQ’s % Agreement with the Dietary Guidelines for Greek Adults ^5^
1	Fruits	480	148.7	30.98%	270.77	56%
2	Vegetables	700	193.82	27.69%	632.63	90%
3	Dairy	334	140.89	42.18%	231.22	69%
4	Legumes	75	14.72	19.63%	2.57	3%
5	Refined Cereals *	220	62.94	29%	132.09	60%
6	Whole Grain Cereals	227	88.05	39%	7.17	3%
7	Red Meat **	19.29	51.22	266%	27.78	144%
8	Poultry	28.93	31.00	107%	11.30	39%
9	Fish	54	23.38	43.30%	6.75	13%
10	Fats & Oils—Nuts	112.5	27.61	24.54%	48.91	44%
11	Drinks & Spirits	45	88.28	130.79%	92.25	205%
12	Sweet	limit	97.62	>100.00%	59.04	>100.00%
13	Fast Food	limit	85.07	>100.00%	33.51	>100.00%
14	Eggs	29	10.52	36.28%	4.76	16%
15	Beverages	500	243.68	48.74%	430.62	86%

^1^ DGG: Dietary Guidelines for Greek Adults. ^2^ Mean intake according to the 24 h recall’s results. ^3^ % agreement of the mean intake that results from the 24 hR and the suggested intake by the Dietary Guidelines for Greek Adults. ^4^ Intake in relation to reported frequency of intake by the FPQ and to reported amount of intake by the 24 hR [frequency of intake (FPQ) × amount of intake (24 hR)].^5^ Percent agreement of combined intake (frequency * 24 hR) and Dietary Guidelines for Greek Adults. * According to the Dietary Guidelines for Greek Adults [27], it is suggested that 447 g of grains should be consumed on a daily basis and most of them should be whole grain cereals. For graphical reasons, we defined this as 220 g of refined cereals and 227 g of whole grain cereals. ** Red meat intake does not include meat added in “fast food”.

## Data Availability

Raw data were generated at [Agricultural University of Athens]. Derived data supporting the findings of this study are available from the corresponding author [AZ] on request.

## Supplementary Materials

The following are available online at https://www.mdpi.com/2072-6643/12/6/1808/s1. Figure S1: Food Propensity Questionnaire (for ages 2 years and more).
