# Supplementary material for: Validation of a Food Propensity Questionnaire for the Hellenic National Nutrition and Health Survey (HNNHS) and Results on This Population’s Adherence to Key Food-Group Nutritional Guidelines
Source: Nutrients. 2020 Jun 17;12(6):1808. doi: 10.3390/nu12061808 (PMC7353208; doi:10.3390/nu12061808)
Supplement: Supplementary file 1 [file nutrients-12-01808-s001.pdf]

VOLUNTEER'S ID	QUESTIONNAIRE'S ID

FOOD PROPENSITY QUESTIONNAIRE Date:____________/________/20

For ages 2 years and more Day/ Month/ Year

Answered by the parent/ legal guardian of the participant.

In the next pages you will find a list of foods divided into groups. Every row corresponds to a specific food or a group of similar foods. In every row, mark ✓ the column that best describes how often you consumed the food item on average, over the past 12 months (of the previous year). Please respond for every food item (row). If you are not sure about how often you consumed a food, select the best possible answer. Avoid leaving unanswered whole pages or sections.

For example, answer separately for white or brown bread. If over the past 12 months you consumed white bread 4-5 times/ every day on average , mark ✓ in the category "4-5 per Day". If you consumed on average 5 slices of brown bread over the year, mark ✓ the category "<1 per Month":

BREAD, CEREALS AND STARCHY PRODUCTS		Never	<	1-3	1	2-4	5-6	1	2-3	4-5	6+
			per Month		per Week			per Day			
Bread, e.g. baked, toast bread, baguette	White									✓	
	Brown		✓								

Seasonality

If you had been consuming some foods more during a specific season of the year, e.g. ice-cream 3 times/ week during summer (3 months), but you had zero consumption for the rest 9 months, you have to calculate the average for 12 months. That means ((3 times per week x 12 weeks)/ 12 months = 3 every month)) that you have to mark ✓ the 1-3 per month column.

MILK PRODUCTS	Never	<1	1-3	1	2-4	5-6	1	2-3	4-5	6+
		per Month		per Week			per Day			
Ice cream or frozen dessert based on milk, e.g. frozen yogurt, milkshake			✓							

Zero consumption

If you never consume a food (e.g. fish eggs), mark ✓ the "Never" column.

FISH AND SEAFOOD	Never	<1	1-3	1	2-4	5-6	1	2-3	4-5	6+
		per Month		per Week			per Day			
Fish eggs	✓									

Abbreviations

E.g.: for example

<1: less than 1

W.: week

6+: 6 or more

Το έντυπο αποτελεί πνευματική ιδιοκτησία του Γεωπονικού Πανεπιστημίου Αθηνών. Κάθε άλλη χρήση, πέρα της προβλεπόμενης για το έργο ΠΑ.ΜΕ.Δ.Υ., απαγορεύεται χωρίς τη γραπτή συγκατάθεση της Αρχής.

Με την συγχρηματοδότηση της Ελλάδας και της Ευρωπαϊκής Ένωσης

www.ygeia-pronoia.gr www.epanad.gov.gr www.espa.gr

For every food (row) of the following list, mark ✓ the column that represents your average consumption during the last 12 months (the last year). If you never consumed this food during the last 12 months mark ✓ the "Never" column.

DAIRY		Never	<1	1-3	1	2-4	5-6	1	2-3	4-5	6+
			per Month		per Week			per Day			
Milk (include milk in cereals and coffee/tea)											
Ice cream or frozen dessert based on milk, e.g. frozen yogurt, milk shake											
Yogurt or yogurt dessert, e.g. yogurt with fruits (include yogurt with cereals)											
Cheese	Smoked										
	Other cheese										

BREAD, CEREALS AND STARCHY PRODUCTS		Never	<1	1-3	1	2-4	5-6	1	2-3	4-5	6+
			per Month		per Week			per Day			
Cereals	Whole wheat, e.g. oat, muesli										
	Other cereals, e.g. made of corn (corn flakes), made of rice										
Bread, e.g. baked, toast bread, baguette	White										
	Brown										
Toasts or rusks	White										
	Brown										
Rice or other dishes with rice, e.g. risotto	White										
	Brown										
Pasta, e.g. macaroni, orzo, noodles	White										
	Brown										
Potatoes	Fried										
	Baked, Boiled or mashed										

FRUITS		Never	<1	1-3	1	2-4	5-6	1	2-3	4-5	6+
			per Month		per Week			per Day			
Dried fruits, e.g. raisins, plums, figs, dates, apricots											
Fruits	Canned, e.g. compote										
	Fresh, π.χ. apple, orange, banana										
Freshly squeezed fruit juice or 100% natural commercial juice											

Το έντυπο αποτελεί πνευματική ιδιοκτησία του Γεωπονικού Πανεπιστημίου Αθηνών. Κάθε άλλη χρήση, πέρα της προβλεπόμενης για το έργο ΠΑ.ΜΕ.Δ.Υ., απαγορεύεται χωρίς τη γραπτή συγκατάθεση της Αρχής.

ΓΕΩΠΟΝΙΚΟ ΠΑΝΕΠΙΣΤΗΜΙΟ ΑΘΗΝΩΝ · Ευρωπαϊκή Ένωση · ΥΠΟΥΡΓΕΙΟ ΥΓΕΙΑΣ www.ygeia-pronoia.gr · Με την συγχρηματοδότηση της Ελλάδας και της Ευρωπαϊκής Ένωσης · ΕΠΙΧΕΙΡΗΣΙΑΚΟ ΠΡΟΓΡΑΜΜΑ ΑΝΑΠΤΥΞΗ ΑΝΘΡΩΠΙΝΟΥ ΔΥΝΑΜΙΚΟΥ www.epanad.gov.gr · ΕΣΠΑ 2007-2013 www.espa.gr

For every food (row) of the following list, mark ✓ the column that represents your average consumption during the last 12 months (the last year). If you never consumed this food during the last 12 months mark ✓ the "Never" column.

		Never	<1	1-3	1	2-4	5-6	1	2-3	4-5	6+
**VEGETABLES**			**per Month**		**per Week**			**per Day**			
Vegetables	Canned, e.g. corn										
	Relish, e.g. pickles										
Green leafy vegetables, e.g. lettuce, spinach, arugula											
Tomato or dressing/ tomato juice											
Cucumber, pepper, pumpkin or eggplant											
Carrot, radish or beetroot											
Onion, garlic or leek											
Cabbage, broccoli, cauliflower or Brussels sprout											
Herbs, e.g. basil, thyme, parsley, fennel											
Artichoke, celery or asparagus											
Mushrooms											
Greens, e.g. notch weeds, chicory, endives											
Peas											
Green beans or ladyfinger											
Legumes, e.g. lentils, beans, lima beans, black-eyed peas, chickpeas, fava beans											
Soya or soya products											

		Never	<1	1-3	1	2-4	5-6	1	2-3	4-5	6+
**EGGS, MEAT AND MEAT PRODUCTS**			**per Month**		**per Week**			**per Day**			
Eggs, e.g. boiled, fried, omelet											
Bush meat, e.g. rabbit, hare, thrush, partridges											
Entrails	Liver										
	Other entrails, e.g. kidneys, intestines, stomach										
Meat products, e.g. ham, turkey slices, sausage, salami, bacon	Smoked										
	Boiled										
Poultry, e.g. chicken, cockerel, turkey, chicken on skewer											

Το έντυπο αποτελεί πνευματική ιδιοκτησία του Γεωπονικού Πανεπιστημίου Αθηνών. Κάθε άλλη χρήση, πέρα της προβλεπόμενης για το έργο ΠΑ.ΜΕ.Δ.Υ., απαγορεύεται χωρίς τη γραπτή συγκατάθεση της Αρχής.

For every food (row) of the following list, mark ✓ the column that represents your average consumption during the last 12 months (the last year). If you never consumed this food during the last 12 months mark ✓ the "Never" column.

EGGS, MEAT AND MEAT PRODUCTS		Never	<1	1-3	1	2-4	5-6	1	2-3	4-5	6+
			per Month		per Week			per Day			
Red meat, e.g. pork, beef, lamb, goat	As main dish, e.g. baked, meat burger, straw										
	In a recipe, e.g. moussakas, pastitsio, ground meat										
	In pie/ bread, e.g. souvlaki, wrap, burger										
Snails											

FISH AND SEAFOOD		Never	<1	1-3	1	2-4	5-6	1	2-3	4-5	6+
			per Month		per Week			per Day			
Canned fish and seafood, e.g. sardine, squid											
Smoked fish, e.g. smoked salmon											
Fresh wide sea fishes (do not include seafood)	Fatty, e.g. mackerel, sardine, chub mackerel, salmon, swordfish, herring										
	Other fishes, e.g. hake, sea bream, goatfish, bogue, pageot										
Freshwater fishes, e.g. trout, perch											
Seafood	shrimps, crawfish or lobster										
	Octopus, squid or cuttlefish										
	Mussels, oysters, scallops or clams										
Fish eggs											

Το έντυπο αποτελεί πνευματική ιδιοκτησία του Γεωπονικού Πανεπιστημίου Αθηνών. Κάθε άλλη χρήση, πέρα της προβλεπόμενης για το έργο ΠΑ.ΜΕ.Δ.Υ., απαγορεύεται χωρίς τη γραπτή συγκατάθεση της Αρχής.

ΓΕΩΠΟΝΙΚΟ ΠΑΝΕΠΙΣΤΗΜΙΟ ΑΘΗΝΩΝ · Ευρωπαϊκή Ένωση · ΥΠΟΥΡΓΕΙΟ ΥΓΕΙΑΣ www.ygeia-pronoia.gr · Με την συγχρηματοδότηση της Ελλάδας και της Ευρωπαϊκής Ένωσης · ΕΠΙΧΕΙΡΗΣΙΑΚΟ ΠΡΟΓΡΑΜΜΑ "ΑΝΑΠΤΥΞΗ ΑΝΘΡΩΠΙΝΟΥ ΔΥΝΑΜΙΚΟΥ" www.epanad.gov.gr · ΕΣΠΑ 2007-2013 www.espa.gr

For every food (row) of the following list, mark ✓ the column that represents your average consumption during the last 12 months (the last year). If you never consumed this food during the last 12 months mark ✓ the "Never" column.

ΝΕΡΟ, ΛΟΙΠΑ ΡΟΦΗΜΑΤΑ ΚΑΙ ΑΛΚΟΟΛ		Never	<1	1-3	1	2-4	5-6	1	2-3	4-5	6+
			per Month		per Week			per Day			
Water	Tap										
	Bottled, e.g. spring water, carbonated, soda										
Beverages (do not include alcoholics)	Low calories ("light")										
	Regular										
Energy drinks, e.g. with caffeine											
Coffee											
Tea	Out of herbs, e.g. chamomile, sage, mountain tea										
	Green, black or white										
Alcohol	Beer										
	Wine, e.g. red, white, rose										
	Spirits, e.g. ouzo, vodka, whiskey,										
	Alcoholic beverages										

OLIVES, FATS AND OILS	Never	<1	1-3	1	2-4	5-6	1	2-3	4-5	6+
		per Month		per Week			per Day			
Olives										
Olive oil										
Other vegetable oils, e.g. sunflower oil, seed oil										
Margarine										
Butter										

SWEETS, BAKED PRODUCTS AND NUTS	Never	<1	1-3	1	2-4	5-6	1	2-3	4-5	6+
		per Month		per Week			per Day			
Honey										
Jam										
Sugar (white or brown) or fructose										
Sweeteners, e.g. aspartame, stevia										
Sugared based fruits served on a teaspoon										
Sesame sweets, e.g. brittle, halvah										

Το έντυπο αποτελεί πνευματική ιδιοκτησία του Γεωπονικού Πανεπιστημίου Αθηνών. Κάθε άλλη χρήση, πέρα της προβλεπόμενης για το έργο ΠΑ.ΜΕ.Δ.Υ., απαγορεύεται χωρίς τη γραπτή συγκατάθεση της Αρχής.

For every food (row) of the following list, mark ✓ to the column that represents your average consumption during the last 12 months (the last year). If you never consumed this food during the last 12 months mark ✓ to the "Never" column.

SWEETS, BAKED PRODUCTS AND NUTS		Never	<1 per Month	1-3 per Month	1 per Week	2-4 per Week	5-6 per Week	1 per Day	2-3 per Day	4-5 per Day	6+ per Day
Chocolate											
Other sweets, e.g. cake, biscuits, croissant, tarts											
Pies	Salty, e.g. cheese pie, spinach pie, ham and cheese pie										
	Sweet, e.g. cream pie										
Pizza											
Nuts, e.g. almonds, walnuts, sunflower seeds, cashew nuts											
Salty snacks, e.g. potato chips, cheese puffs, pop corn											

In the following section please select the right answer for you, by marking ✓ to the circle located on the left of your answer.

How often do you consume organic products?

◯ always ◯usually ◯sometimes ◯rarely ◯never ◯I don't know

How often do you fry the fish that you consume?

◯ always ◯usually ◯sometimes ◯rarely ◯never ◯I don't know ◯I don't eat fish

How often do you eat other fried foods at home?

◯ less than 1 time per week ◯1-3 times per week. ◯4-6 times per week ◯every day ◯never

How often do you eat fried foods away from home or you order takeaway?

◯ less than 1 time per week ◯1-3 times per week. ◯4-6 times per week ◯every day ◯never

What kind of fat/oil do you use to fry/pan-fry/cook at home?

◯ butter ◯margarine ◯olive oil ◯other vegetable oil ◯I don't know
◯ I don't cook at home

How often do you consume homemade sweets?

◯ always ◯usually ◯sometimes ◯rarely ◯never ◯I don't know

Which is the main kind of fat/oil that you use for homemade sweets?

◯ butter ◯margarine ◯olive oil ◯other vegetable oil ◯I don't know

Write the butter's or margarine's company name and product name that you usually use:

◯I don't know

How often do you eat homemade pies?

◯ always ◯usually ◯sometimes ◯rarely ◯never ◯I don't know

Do you add salt in your food when on table or during the cooking?

◯ never/nowhere ◯cooking ◯table ◯table and cooking

Who usually cooks at home?

◯ myself ◯wife-husband/partner ◯mother/father ◯grandmother/grandfather ◯else
◯ I don't eat at home

Το έντυπο αποτελεί πνευματική ιδιοκτησία του Γεωπονικού Πανεπιστημίου Αθηνών. Κάθε άλλη χρήση, πέρα της προβλεπόμενης για το έργο ΠΑ.ΜΕ.Δ.Υ., απαγορεύεται χωρίς τη γραπτή συγκατάθεση της Αρχής.

**Are they any other foods that you usually consume <u>at least once a week?</u> (Do not include any of the foods that has been included in the previous pages)**	Other foods that you usually eat at least once a week
	(a)
	(b)
	(c)

- Who answered the questionnaire?

  ☐ Myself ☐ Mother ☐ Father ☐ Else, define

- How much time did you spend to complete the questionnaire?

  ☐☐ Minutes

Your comments will help us very much. Please add in the section below, any comment or note that you have about the questionnaire and its completion.

WE SINCERELY THANK YOU!

Το έντυπο αποτελεί πνευματική ιδιοκτησία του Γεωπονικού Πανεπιστημίου Αθηνών. Κάθε άλλη χρήση, πέρα της προβλεπόμενης για το έργο ΠΑ.ΜΕ.Δ.Υ., απαγορεύεται χωρίς τη γραπτή συγκατάθεση της Αρχής.

ΓΕΩΠΟΝΙΚΟ ΠΑΝΕΠΙΣΤΗΜΙΟ ΑΘΗΝΩΝ
Ευρωπαϊκή Ένωση
ΥΠΟΥΡΓΕΙΟ ΥΓΕΙΑΣ
www.ygeia-pronoia.gr
Με την συγχρηματοδότηση της Ελλάδας και της Ευρωπαϊκής Ένωσης
ΕΠΙΧΕΙΡΗΣΙΑΚΟ ΠΡΟΓΡΑΜΜΑ ΑΝΑΠΤΥΞΗ ΑΝΘΡΩΠΙΝΟΥ ΔΥΝΑΜΙΚΟΥ
www.epanad.gov.gr
ΕΣΠΑ 2007-2013
www.espa.gr
